# Spermidine cures yeast of prions

**DOI:** 10.15698/mic2016.01.474

**Published:** 2015-12-25

**Authors:** Shaun H. Speldewinde, Chris M. Grant

**Affiliations:** 1 University of Manchester, Faculty of Life Sciences, The Michael Smith Building, Oxford Road, Manchester, M13 9PT, UK.

**Keywords:** autophagy, oxidative stress, prions, spermidine, yeast

## Abstract

Prions are self-perpetuating amyloid protein aggregates which underlie various neurodegenerative diseases in mammals. The molecular basis underlying their conversion from a normally soluble protein into the prion form remains largely unknown. Studies aimed at uncovering these mechanism(s) are therefore essential if we are to develop effective therapeutic strategies to counteract these disease-causing entities. Autophagy is a cellular degradation system which has predominantly been considered as a non-selective bulk degradation process which recycles macromolecules in response to starvation conditions. We now know that autophagy also serves as a protein quality control mechanism which selectively degrades protein aggregates and damaged organelles. These are commonly accumulated in various neurodegenerative disorders including prion diseases. In our recent study [Speldewinde *et al. *Mol. Biol. Cell. (2015)] we used the well-established yeast [*PSI*^+^]/Sup35 and [*PIN¬^+^*]/Rnq1 prion models to show that autophagy prevents sporadic prion formation. Importantly, we found that spermidine, a polyamine that has been used to increase autophagic flux, acts as a protective agent which prevents spontaneous prion formation.

The molecular basis by which prions arise spontaneously is poorly understood. Our data indicate that oxidative protein damage to Sup35, which is a known trigger for *de novo* prion formation, is normally suppressed by autophagy. Oxidatively damaged Sup35 was found to accumulate in mutants lacking core components of the autophagy pathway, and this was found to correlate with an increased frequency of *de novo *[*PSI*^+^] prion formation. We showed that growth under anaerobic conditions in the absence of molecular oxygen prevented the accumulation of oxidized Sup35 and abrogated the high frequency of [*PSI*^+^] formation in an autophagy mutant. This suggests that autophagy normally functions to clear oxidatively damaged proteins prior to their conversion to the prion form. A protective role for autophagy in preventing *de novo *prion formation was further confirmed by showing that increasing autophagic flux by treatment with spermidine abrogates the formation of prions in mutants which normally show high rates of *de novo* prion formation. This important new finding strongly implicates autophagy as a defense system which protects against oxidative damage of the non-prion form of a protein. This is an important trigger for the formation of the heritable prion conformation, an event that has also been implicated in the formation of mammalian prions.

Our study highlights the potential use for autophagy-inducing agents such as spermidine in the prevention of the very early stages of spontaneous prion formation i.e. effectively acting as a prion prevention agent (Fig. 1). By improving the clearance of damaged/misfolded proteins, there is less possibility for these abnormal proteins to accumulate and to act as nucleation sites catalyzing the aggregation of other damaged proteins. Polyamines such as spermidine are polycations which play multiple roles in cell growth, proliferation and longevity. Their beneficial effects in prolonging lifespan are thought to be mediated by increasing autophagic flux. Spermidine inhibits histone acetylases and the resulting alterations in the acetylproteome increases the transcription of different autophagy-related genes (Fig. 1). More work will be required to determine whether the abrogation of prion formation by spermidine solely depends on increasing autophagic flux, or whether spermidine additionally promotes other stress protective pathways. For example, spermidine supplementation has been linked with increased stress tolerance including heat and oxidative stress, which is not only mediated by increasing autophagic flux. Whether spermidine modulates the expression of other stress responsive genes, such as heat shock and antioxidant genes which are known to influence protein misfolding and prion formation, has not been fully established. Hence, spermidine may amelioarte prion fomation via multiple mechnisms including the induction of autophagy and other stress-related pathways. 

**Figure 1:**
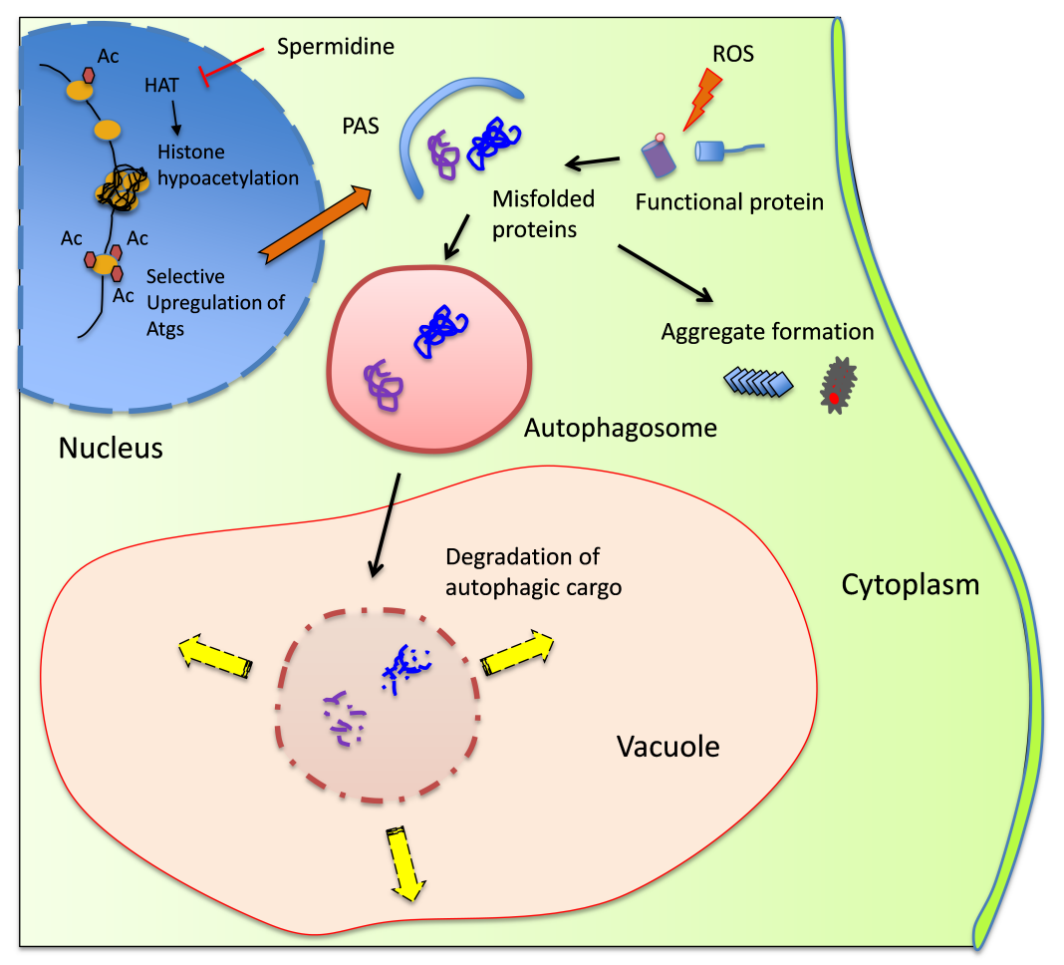
FIGURE 1: Model illustrating the cytoprotective action of spermidine in preventing spontaneous prion formation. Spermidine inhibits the activity of histone acetyl transferases (HAT) that function to insert acetyl (Ac) groups on histone H3. This causes global gene silencing, but certain genes including autophagy-related genes (Atgs) remain acetylated thus inducing autophagic activity. Reactive oxygen species (ROS), whether endogenous or exogenous, may damage soluble proteins leading to their misfolding/aggregation. These abnormal proteins can be encapsulated by the pre-autophagosomal structure (PAS), which then fully matures into an autophagosome. The autophagosome with its cargo fuses with the vacuole where resident hydrolases can degrade the cargo and the resultant products can be channeled for biosynthesis or for energy generation. Inducing autophagy by spermidine treatment prevents prion formation by removing misfolded/oxidized proteins prior to their conversion to the prion form.

Recent data from our lab, which was not included in the original pulication, demonstrates that spermidine treatment can also promote the clearance of Sup35 aggregates from cells in an autophagy-dependent manner (Fig. 2). We used a Sup35NM-GFP fusion construct to visualize Sup35 aggregate formation in [*PSI*^+^]-versions of wild-type and *atg1 *mutant strains. Following short-term induction of the Sup35NM-GFP fusion construct, fluorescent foci can be detected due to the coalescence of newly made Sup35NM-GFP with pre-existing Sup35 aggregates. Fluorescent Sup35 aggregates are normally visible in approximately 70% of [*PSI*^+^] cells examined (Fig. 2). We found that spermidine treatment reduced this number such that visible Sup35 aggregates are only detected in approximately 25% of cells. This did not occur in an *atg1 *mutant confirming the requirement for an active autophagy pathway to clear aggregates in response to spermidine treatment (Fig. 2). This experiment suggests that increasing autophagic flux via spermidine treatment, not only promotes the removal of smaller misfolded/oxidized Sup35 proteins, but can also promote the removal of larger molecular weight Sup35 aggregates which are already formed within cells. It should be emphasized that visible Sup35-GFP aggregates do not necessarily correspond to the number of true heritable [*PSI*^+^] aggregates in cells and more work will be required to examine whether spermidine treatment can really cure cells of the [*PSI*^+^] prion. This may be unlikely though, since the presence of only a few low molecular weight Sup35 propagons will be inherited by daughter cells resulting in [*PSI*^+^] prion transmission. However spermidine may well be beneficial in the treatment of other non-heritable and amorphous protein aggregate diseases. 

**Figure 2:**
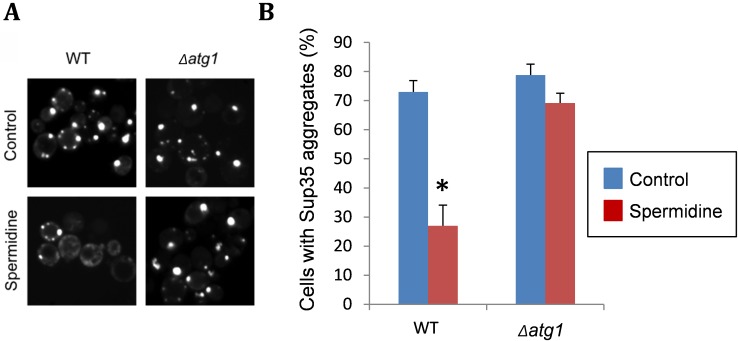
FIGURE 2: Spermidine treatment decreases the number of cells with visible Sup35 fluorescent aggregates. **(A) **Representative fluorescence micrographs are shown for [*PIN*^+^][*PSI*^+^] versions of the wild-type yeast strain 74D-694 (*MATa ade1-14 ura3-52 leu2-3,112 trp1-289 his3-200)* and an isogenic *atg1 *mutant containing the Sup35NM-GFP plasmid. Strains were grown in minimal media in the presence or absence of 4 mM spermidine for 48 hours to induce autophagy. Sup35NM-GFP was induced with copper for one hour. Following copper induction, fluorescent foci can be detected due to the coalescence of newly made Sup35NM-GFP with pre-existing Sup35 aggregates. **(B) **The percentage of cells containing visible puncta is shown for each strain from an average of 300 cells counted. Data shown are the means of three independent biological repeat experiments ± SD. The number of visible aggregates in the wild-type strain treated with spermidine is significantly different to the number of aggregates detected in the same strain in the absence of spermidine (*p = <0.001).

Polyamines, such as spermidine, are present in millimolar quantities within all eukaryotic cells. They play essential roles in a multitude of cellular processes related to cell growth, proliferation and metabolism. Spermidine is a naturally occurring polyamine and rich dietary sources include soy products, legumes, corn, and whole grain cereals. The cellular levels of polyamines, such as spermidine, decline with age and have been linked to lifespan and age-related disorders. Supplementation of spermidine into dietary regimes may therefore have added benefits for cellular health and healthy ageing. Relative to other established pharmacological inducers of autophagy, such as rapamycin and resveratrol, spermidine can be readily obtained from dietary sources and does not exhibit deleterious side effects. This places spermidine as a promising therapeutic agent for the prevention and amelioration of protein homeostasis and related aggregation diseases.

